# Robustness Comparison of the Virtual Bolus Method and Robust Optimization in Postmastectomy Radiation Therapy Using Volumetric Modulated Arc Therapy

**DOI:** 10.7759/cureus.105492

**Published:** 2026-03-19

**Authors:** Ryohei Yamauchi, Fumihiro Tomita, Yujiro Nakajima, Yukio Fujita, Naoki Tohyama, Norifumi Mizuno, Satoshi Ishikura

**Affiliations:** 1 Department of Radiation Oncology, St. Luke’s International Hospital, Tokyo, JPN; 2 Department of Radiological Sciences, Komazawa University, Tokyo, JPN; 3 Department of Radiation Oncology, Saitama Medical Center, Saitama Medical University, Kawagoe, JPN

**Keywords:** breast cancer, intensity-modulated radiation therapy, respiratory motion, robust planning, virtual bolus

## Abstract

Background

In postmastectomy radiation therapy (PMRT), respiratory motion compromises dose delivery. Quantitative comparisons between virtual bolus (VB) and robust optimization (RO) remained limited, and there is no definitive consensus regarding optimal skin-flash strategies. This study evaluated the relative motion robustness of VB and RO in PMRT and explored the influence of VB thickness and density on dosimetric robustness.

Methodology

This study included 20 patients with left-sided PMRT planned in RayStation using standard optimization (SO), VB (27 combinations of three planning target volume (PTV) margins, three VB thicknesses, and three densities), and RO (three uncertainty settings). Respiratory motion was simulated by shifting the isocenter by 3-15 mm. Robustness was quantified using changes in chest wall D_98%_ and D_2%_ relative to the nominal plan.

Results

VB configurations showed substantial variability. The most robust plans consistently involved a VB thickness equal to the PTV margin plus 8 mm at 0.4 g/cm³. Under a 5-mm shift, median D_2%_ changes for SO, VB, and RO were 2.6%, −0.8%, and 0.7%, while D_98%_ changes were −8.7%, −0.3%, and −2.6%, respectively. Top-ranked VB settings maintained dose stability even when motion exceeded the applied PTV margin. Conversely, thin, low-density VB configurations caused marked D_2%_ escalation (up to 3%) and were considered suboptimal. For representative configurations, changes in heart D_mean_ and lung V_20Gy_ under isocenter shifts were small and comparable among SO, VB, and RO.

Conclusions

Under the tested conditions, appropriately configured VB demonstrated greater dosimetric robustness of target coverage than RO and SO. Robustness relied on the combined effects of PTV margin, VB thickness, and density. A configuration with VB thickness equal to the PTV margin plus 8 mm at 0.4 g/cm³ achieved the most stable performance, providing practical dosimetric insight for robustness-oriented PMRT planning.

## Introduction

Volumetric modulated arc therapy (VMAT) has become popular for postmastectomy radiation therapy (PMRT) due to its superior target coverage and organs at risk (OARs) sparing compared with three-dimensional conformal radiotherapy [1-8]. Respiratory motion [4,9], tissue swelling, and anatomical deformation [10-13] reduce target coverage in PMRT. Therefore, ensuring dose stability despite these effects is essential. To avoid underdosage in the build-up region, the International Commission on Radiation Units and Measurements (ICRU) Report 83 recommends ensuring sufficient fluence beyond the skin surface (“skin-flash”) [14].

In clinical practice, two conceptually different strategies, the virtual bolus (VB) and robust optimization (RO) methods, are used to achieve skin-flash in modulated breast radiotherapy [2,15]. The VB method explicitly expands the superficial optimization region by adding an artificial structure of specified thickness and density outside the planning target volume (PTV). This forces fluence beyond the skin surface during optimization. After optimization, the VB is removed, and the final dose is calculated on the original computed tomography (CT), thereby preserving fluence extension beyond the skin. In contrast, RO directly incorporates setup and motion uncertainties into the objective function but does not inherently promote fluence extension into air regions. These mechanisms differ fundamentally; thus, their impact on motion robustness remains unclear.

Previous studies have provided partial insights into these methods [2,3,12,13,16-21]. Lizondo et al. demonstrated that VB densities of approximately −400 to −500 HU maintain robustness, whereas Rossi et al. revealed that VB thickness affects stability under deformation [3,13]. RO has provided superior robustness to VB using water-equivalent density in accelerated partial breast irradiation [18]. However, no PMRT study has systematically compared VB and RO under equivalent motion scenarios or investigated the combined influence of VB thickness and density, thereby limiting evidence-based selection of skin-flash strategies.

To address these gaps, we systematically assessed 27 VB configurations to characterize the combined effects of VB thickness and density. We then compared the robustness of VB and RO plans, defined as the preservation of target dose coverage under geometric uncertainty, utilizing perturbation-based dose calculations simulating respiratory motion. This study aimed primarily to evaluate whether VB or RO provides greater robustness in PMRT and secondarily to identify VB thickness-density combinations associated with stable target dose preservation.

## Materials and methods

Study population

This study retrospectively enrolled 20 patients with left-sided breast cancer who underwent PMRT without reconstruction at our institution from November 2020 to November 2022. Patient characteristics are summarized in Table 1. The Institutional Review Board of our institution approved the study protocol (approval number: 22-R009).

**Table 1:** Patient characteristics. BMI: body mass index; CTV: clinical target volume; CW: chest wall; IMC: internal mammary chain; Sc: supraclavicular fossa

Patient number	Age (years)	BMI (kg/m²)	CTV_Sc volume (cm^3^)	CTV_CW volume (cm^3^)	CTV_IMC volume (cm^3^)
1	45	29.5	64.4	485.2	20.9
2	59	21.2	104.4	278.3	3.35
3	52	32.8	149.1	878.0	11.5
4	54	21.7	68.0	457.5	12.3
5	50	26.7	92.7	377.1	6.6
6	58	21.3	86.0	306.7	12.8
7	62	21.4	74.3	392.6	4.9
8	34	21.0	29.1	222.0	6.5
9	55	19.1	44.5	200.0	5.6
10	43	21.4	50.0	180.5	10.8
11	47	29.0	18.1	179.4	6.8
12	60	19.4	53.4	220.4	13.1
13	37	22.9	111.2	411.7	1.8
14	46	23.5	75.1	491.6	4.8
15	42	22.9	97.8	364.7	5.2
16	49	25.4	146.9	318.5	3.4
17	39	20.2	63.9	110.1	7.4
18	61	27.6	148.7	500.6	15.0
19	61	25.6	79.6	321.5	9.4
20	36	16.1	70.7	255.3	4.7

Delineation for target and OARs

A SOMATOM Confidence RT Pro scanner (Siemens Healthcare, Erlangen, Germany) with 2-mm slice thickness was used to acquire planning CT images. Patients were immobilized supine with both arms raised using a vacuum cushion and wing board (CIVCO Radiotherapy, IA, USA).

An experienced radiation oncologist delineated the clinical target volume (CTV), following consensus guidelines [22]. In all cases, the CTVs included the chest wall (CW), lymph nodes around the supraclavicular fossa (SC), and the internal mammary chain (IMC). Each CTV (CW, SC, and IMC) was expanded isotropically by 5 mm to generate the initial PTV, followed by clinically standard cropping near the heart and lungs.

PTV margin directly interacts with VB thickness; thus, additional PTV_CW variants (3-, 5-, and 8-mm expansions) were intentionally generated for the CW only. This permitted assessment of how VB performance depends on the underlying PTV size. A cropped PTV (PTVcropped) was defined to assess dose coverage for all PTVs, excluding the outer 3 mm from the skin surface and 3 mm from the heart and lungs. OARs, including the heart, contralateral breast, bilateral lungs, and esophagus, were contoured following atlas guidelines [23].

VMAT planning using standard optimization

The standard optimization (SO) VMAT plan, developed without robust considerations, served as the reference for comparison with the VB and RO methods. Optimization was performed on PTVopt, defined as the PTV excluding the outer 3 mm from the skin surface to prevent over-compensation in the build-up region.

All plans were generated in RayStation 10ASP1 (RaySearch Laboratories, Stockholm, Sweden) for delivery on a TrueBeam 6 MV (Varian Medical Systems, Palo Alto, CA, USA). Four split partial arcs were used to create VMAT plans, following the approach proposed by Boman et al. [1], with optimized gantry angles for target coverage and OAR sparing. Dose calculation employed a collapsed-cone convolution algorithm with heterogeneity correction and a 2-mm grid. The prescribed dose was 50 Gy in 25 fractions to PTVcropped D_50%_.

A standardized optimization template was utilized for all patients (Table 2). To minimize operator dependence, each plan underwent three consecutive optimization runs using identical objectives and weights. No manual adjustments were made during optimization.

**Table 2:** Optimization objectives and constraints used for VMAT planning. All optimization functions were identical across planning strategies unless otherwise specified. The ROI labeled PTV_CW* corresponds to different structures depending on the planning approach: PTVopt for the standard optimization plan, PTV_CW for the virtual bolus plan, and CTV_CW for the robust optimization plan. The Robust column indicates structures for which robust optimization was enabled in the robust optimization plans. CTV: clinical target volume; CW: chest wall; DVH: dose-volume histogram; EUD: equivalent uniform dose; IMC: internal mammary chain; PTV: planning target volume; ROI: region of interest; Sc: supraclavicular; VMAT: volumetric modulated arc therapy

Function	ROI	Description	Robust	Weight
Min dose	CTV_SC	Minimum dose 4,950 cGy	★	200.00
Max dose	CTV_SC	Maximum dose 5,050 cGy	★	200.00
Min dose	PTV_CW *	Minimum dose 4,950 cGy	★	200.00
Max dose	PTV_CW *	Maximum dose 5,050 cGy	★	200.00
Min dose	CTV_IMC	Minimum dose 4,950 cGy	★	200.00
Max dose	CTV_IMC	Maximum dose 5,050 cGy	★	200.00
Max dose	Heart	Maximum dose 4,000 cGy		20.00
Max DVH	Heart	Maximum DVH 3,000 cGy to 2.00% volume		20.00
Max DVH	Heart	Maximum DVH 2,000 cGy to 10.00% volume		20.00
Max EUD	Heart	Maximum EUD 500 cGy, Parameter A1		20.00
Max DVH	Lung	Maximum DVH 2,000 cGy to 18.00% volume		20.00
Max DVH	Lung	Maximum DVH 500 cGy to 50.00% volume		20.00
Max dose	Cord	Maximum dose 2,500 cGy		20.00
Max EUD	Esophagus	Maximum EUD 2,000 cGy, Parameter A1		20.00
Max dose	Trachea	Maximum dose 4,000 cGy		20.00
Max dose	Thyroid	Maximum dose 4,000 cGy		20.00
Max EUD	Brachial_Head	Maximum EUD 2,500 cGy, Parameter A1		20.00
Max dose	Brachial_Plex	Maximum dose 5,250 cGy		20.00
Max dose	Contralateral Breast	Maximum dose 2,000 cGy		20.00
Max DVH	LtLung	Maximum DVH 2,000 cGy to 40.00% volume		20.00
Max DVH	RtLung	Maximum DVH 500 cGy to 35.00% volume		20.00
Max dose	External	Maximum dose 5,050 cGy		100.00
Dose fall-off	External	[H]5,000 cGy [L]4,500 cGy, low-dose distance 0.20 cm		40.00
Dose fall-off	External	[H]5,000 cGy [L]2,000 cGy, low-dose distance 1.50 cm		40.00

VMAT planning using the VB method

**Figure 1:**
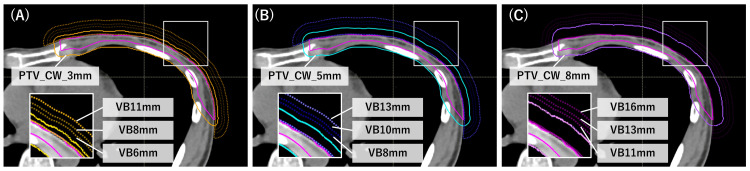
Example of the relationship between VB configurations and PTV margins. Each panel illustrates the relationship between the clinical target volume of the chest wall (CTV_CW), planning target volume of the chest wall (PTV_CW), and virtual bolus (VB) structures. Panels (A), (B), and (C) correspond to PTV_CW margins of 3, 5, and 8 mm, respectively. Solid pink lines indicate CTV_CW, solid colored lines indicate PTV_CW, and dotted lines represent VB structures. VB structures were generated by adding 3, 5, and 8 mm beyond each PTV_CW and are labeled by their total thickness.

Figure 1 illustrates that nine VB structures were generated by adding three different VB margins (3, 5, and 8 mm) to the PTV_CW and subtracting the result from the external contour. Three physical densities (0.4, 0.7, and 1.0 g/cm³) were applied for each of the three VB geometries, which led to 27 combinations. An effective VB thickness was defined as the sum of the PTV and VB margins. These settings were selected based on previous studies and supplemented with adjacent values to cover a clinically realistic range [2,3,13,16,18-21]. The inclusion of these clinically representative values permitted direct comparison with conventional planning practices and previously published benchmark conditions.

VB plans were developed using identical beam arrangements and optimization objectives as the SO plans. The VB structure was applied only during optimization to enable fluence extension beyond the skin, then removed before final dose calculation on the original CT while maintaining identical monitor units and multileaf collimator positions. A total of 540 VB plans (27 configurations × 20 patients) were generated. Plans were labeled as “PTV_X_mm/VB_Y_mm,” where X denotes the PTV_CW margin, and Y indicates the VB thickness.

VMAT planning using RO

The RO module integrated in RayStation was used to develop RO plans. This module adopts a minimax optimization framework that minimizes objective-function penalties under worst-case scenarios. Anisotropic position uncertainties (3, 5, or 8 mm for anterior/left directions and 5 mm for other directions) were applied to each CTV in the optimization settings to ensure consistent comparison with the VB method. These values were selected to correspond with the PTV margins used in the VB approach. For each RO plan, 7-12 uncertainty scenarios were considered, including the nominal scenario and positional shifts along the six cardinal directions, depending on the selected uncertainty magnitude. RO objectives were applied to the CTVs to maintain target coverage across all scenarios. Beam geometry, optimization goals, and dose prescription were identical to the SO and VB plans to ensure fair comparison.

Robustness analysis across SO, VB, and RO plans

The built-in perturbation module in RayStation was used to generate perturbed dose distributions. The isocenter was systematically shifted along the posterior direction by 3, 5, 8, 10, and 15 mm. This posterior shift simulates the relative anterior displacement of the CW due to inhalation or swelling. Respiratory motion involves superior-inferior displacement, whereas the anterior limitation of the fluence is the primary dosimetric concern. Therefore, anterior displacement was selected as a representative worst-case geometric scenario for target coverage degradation. The relative differences in CTV_CW D_98%_ and D_2%_ were calculated against the unshifted condition for each perturbed plan. Robustness was quantified using (1) the magnitude of these dose differences and (2) the shift distance needed to produce a 3% change, estimated by linear interpolation. The latter provided a supplementary indicator of motion tolerance. For OARs, the relative differences in the mean dose (D_mean_) for the heart and the V_20Gy_ for the lungs were evaluated under the same perturbation conditions.

Robustness ranking and inter-method comparison

All VB configurations for each PTV margin (3, 5, and 8 mm) were ranked based on the D_98%_ and D_2%_ differences obtained under the corresponding isocenter shift. Configurations with smaller dose deviations were assigned to better ranks.

Margin-specific ranks were then converted to numerical scores (1 for best and 9 for worst) and summed across the three margins to identify the overall robustness. This scoring approach enabled the identification of VB settings that were consistently stable across different PTV sizes.

According to this aggregated ranking, three representative VB configurations were selected for inter-method comparison with SO and RO: (1) the top-ranked VB setting, representing a configuration with consistently high dose stability; (2) the VB configuration recommended by Lizondo et al., serving as a literature-based benchmark; and (3) a water-equivalent VB configuration (density, 1.0 g/cm³), indicating a commonly employed clinical practice. Relative D_98%_ and D_2%_ differences at 3-, 5-, and 8-mm shifts were compared among SO, VB, and RO.

Statistical analysis

Overall differences among planning methods (SO, VB, and RO) were investigated using the Friedman test, followed by Bonferroni-corrected Wilcoxon signed-rank tests for pairwise inter-method comparisons. A p-value <0.05 indicated statistical significance. EZR (version 2.4-0) was used for analyses.

## Results

Robustness evaluation across SO, VB, and RO plans

**Figure 2:**
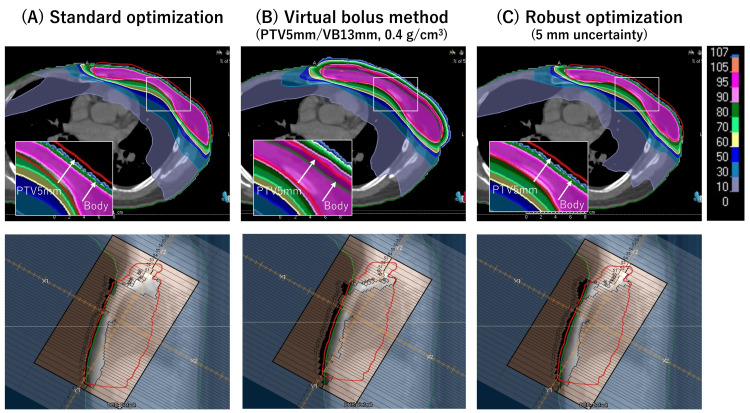
Representative beam’s eye view and axial dose distribution for the three planning strategies. Representative beam’s eye view (top row) and axial dose distributions (bottom row) are shown for (A) standard optimization, (B) virtual bolus-based planning (planning target volume (PTV) margin 5 mm with a VB thickness of 13 mm at a density of 0.4 g/cm³), and (C) robust optimization with a 5-mm positional uncertainty. The green contour indicates the body surface, and the red contour represents the PTV with a 5-mm margin.

VB plans demonstrated clear fluence extension beyond the skin surface, whereas SO plans showed almost no flash, and RO plans showed an intermediate pattern (Figure 2). These observations visually validate the expected differences in fluence behavior among the three methods.

**Figure 3:**
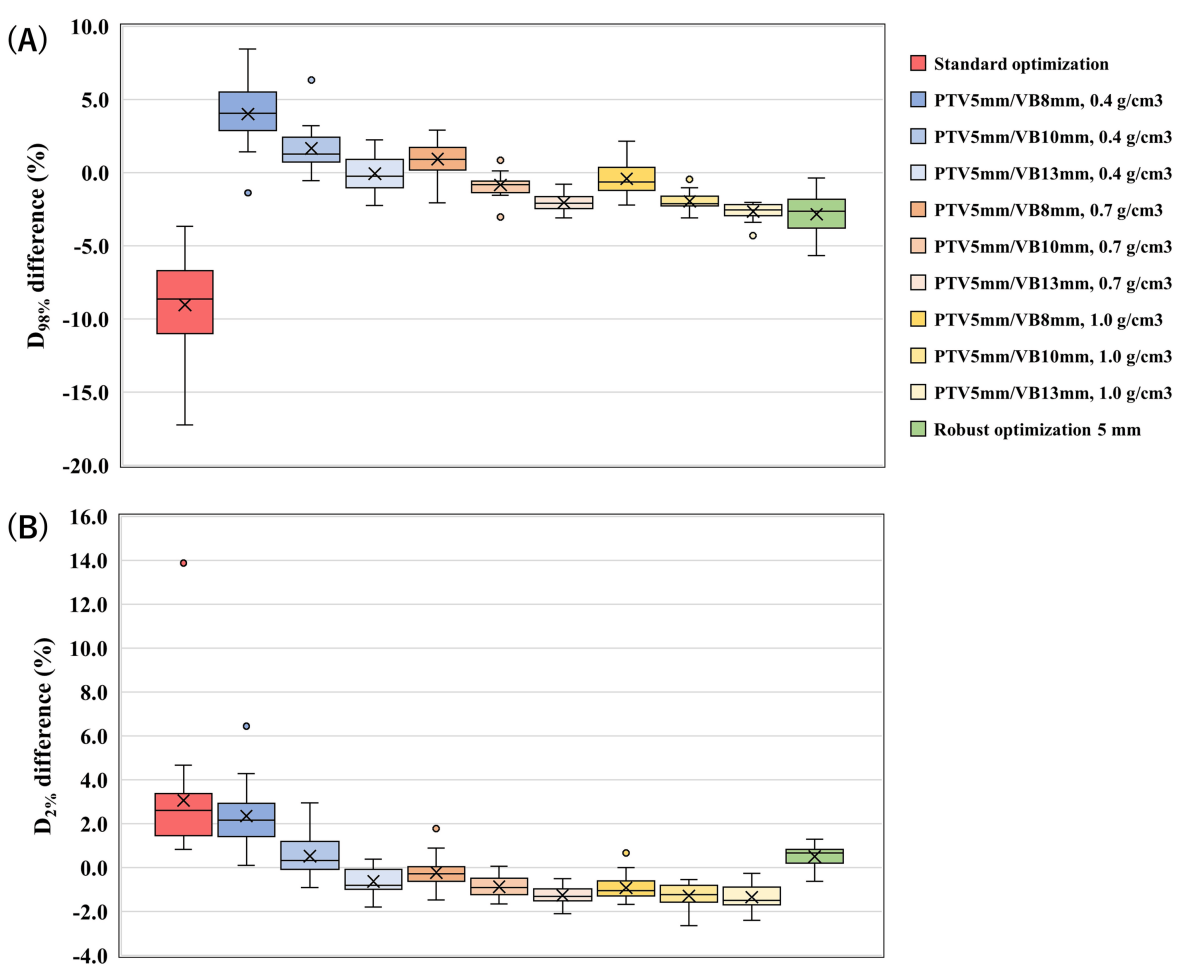
Changes in D98% and D2% under a 5-mm isocenter shift. Panels (A) and (B) show relative changes in CTV_CW D_98%_ and D_2%_, respectively. Positive values indicate an increase in dose, while negative values indicate a decrease. Boxplots represent the median and interquartile range, with whiskers indicating the full range. CTV: clinical target volume; CW: chest wall

Under a 5-mm isocenter shift (Figure 3), SO demonstrated the greatest degradation, whereas RO exhibited intermediate changes. VB plans generally exhibited smaller dose deviations; however, their robustness varied depending on VB thickness and density.

Furthermore, ranking of all 27 VB configurations identified the setting with VB thickness equal to PTV margin plus 8 mm at 0.4 g/cm³ as the most robust across all margins. This configuration corresponds to the PTV5mm/VB13mm, 0.4 g/cm³ plan illustrated in Figure 3. Thin-thickness configurations (PTV margin plus 3 mm) combined with higher density (0.7-1.0 g/cm³) represented the next-best group, thereby confirming that robustness depends on the interaction between VB thickness and density.

Table 3 summarizes representative inter-method comparisons. Under a 5-mm isocenter shift, the top-ranked VB plan (VB thickness equal to PTV margin plus 8 mm at 0.4 g/cm³) demonstrated minimal dose changes (D_98%_ = −0.3%, D_2%_ = −0.8%), which significantly outperform RO (D_98%_ = −2.6%, D_2%_ = 0.7%) and SO (D_98%_ = −8.7%, D_2%_ = 2.6%) (p < 0.001).

**Table 3:** Changes in D98% and D2% under 3-, 5-, and 8-mm isocenter shifts. Values represent median (interquartile range (IQR)) percentage changes relative to the original plan. Negative values indicate dose reduction and positive values indicate dose increase. PTV: planning target volume; RO: robust optimization; SO: standard optimization; VB: virtual bolus

Planning strategy	3 mm shift (PTV margin = 3 mm for VB; uncertainty = 3 mm for RO)		5 mm shift (PTV margin = 5 mm for VB; uncertainty = 5 mm for RO)		8 mm shift (PTV margin = 8 mm for VB; uncertainty = 8 mm for RO)	
	D_98%_ difference (%)	D_2%_ difference (%)	D_98%_ difference (%)	D_2%_ difference (%)	D_98%_ difference (%)	D_2%_ difference (%)
SO	−3.9 (−4.8-−3.0)	1.0 (0.6-1.7)	−8.7 (−10.8-−6.8)	2.6 (1.6-3.3)	−19.7 (−23.9-−17.2)	4.7 (2.9-5.5)
VB (PTV margin +8 mm, 0.4 g/cm³)	−0.4 (−0.7-0.1)	−0.5 (−0.7-−0.2)	−0.3 (−1.0-0.8)	−0.8 (−1.0-−0.1)	−0.1 (−0.7-0.3)	−0.6 (−1.0-0.0)
VB (PTV margin +5 mm, 0.4 g/cm³)	0.6 (0.2-1.4)	0.1 (−0.1-0.3)	0.9 (0.2-1.7)	0.3 (0.0-1.2)	1.9 (1.1-3.4)	0.5 (0.3-1.3)
VB (PTV margin +5 mm, 1.0 g/cm³)	−1.3 (−1.7-−1.0)	−0.9 (−1.0-−0.7)	−2.1 (−2.3-−1.6)	−1.2 (−1.6-−0.8)	−2.9 (−3.6-−1.9)	−1.9 (−2.6-−1.3)
RO	−2.1 (−2.7-−1.4)	0.5 (0.3-0.6)	−2.6 (−3.7-−2.0)	0.7 (0.2-0.8)	−3.5 (−4.1-−2.7)	0.5 (0.1-0.8)

For OARs, heart D_mean_ and lung V_20Gy_ were evaluated under 3-, 5-, and 8-mm isocenter shifts for representative VB configurations, SO, and RO (Table 4). Overall, OAR dose differences relative to the nominal plan were comparable among the three planning strategies within the evaluated conditions, with no systematic increase observed for VB plans compared with SO or RO across the assessed shift magnitudes.

**Table 4:** Changes in organ-at-risk dose parameters under isocenter shifts for representative planning strategies. Values represent median (interquartile range (IQR)) percentage changes relative to the original plan. Positive values indicate dose increase. PTV: planning target volume; RO: robust optimization; SO: standard optimization; VB: virtual bolus

Planning strategy	3 mm shift (PTV margin = 3 mm for VB; uncertainty = 3 mm for RO)		5 mm shift (PTV margin = 5 mm for VB; uncertainty = 5 mm for RO)		8 mm shift (PTV margin = 8 mm for VB; uncertainty = 8 mm for RO)	
	Heart D_mean_	Lung V_20Gy_	Heart D_mean_	Lung V_20Gy_	Heart D_mean_	Lung V_20Gy_
SO	15.3 (14.0-17.3)	7.3 (6.9-9.2)	27.8 (25.1-31.4)	12.1 (11.4-15.1)	49.7 (43.9-56.0)	19.0 (17.8-23.3)
VB (PTV margin +8 mm, 0.4 g/cm³)	15.7 (14.3-18.0)	7.1 (6.5-8.1)	28.4 (25.6-31.9)	11.0 (10.6-12.6)	51.6 (44.7-58.6)	17.9 (15.1-19.6)
VB (PTV margin +5 mm, 0.4 g/cm³)	15.6 (14.0-17.1)	7.4 (6.1-8.2)	27.9 (25.2-31.7)	11.1 (10.5-13.2)	49.8 (45.3-54.3)	16.3 (15.4-19.7)
VB (PTV margin +5 mm, 1.0 g/cm³)	16.3 (14.4-18.7)	7.3 (6.3-8.2)	28.9 (25.5-32.5)	11.2 (10.3-12.8)	53.6 (46.3-59.8)	17.7 (16.4-20.0)
RO	16.4 (14.8-19.5)	7.2 (6.3-8.4)	30.0 (25.6-33.6)	12.2 (10.5-13.8)	52.0 (44.7-61.4)	18.9 (16.7-21.3)

Association between perturbed dose and isocenter shift

**Figure 4:**
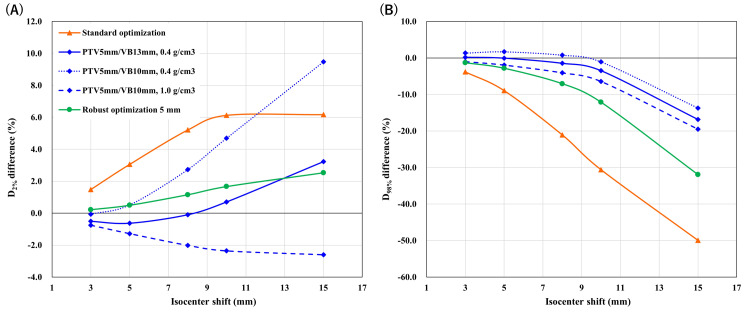
Dose–shift relationships for the three planning strategies. Panels (A) and (B) show relative changes in CTV_CW D_98%_ and D_2%_, respectively, as a function of isocenter shift distance. Lines represent standard optimization, selected virtual bolus–based plans with a 5-mm PTV margin, and robust optimization with a 5-mm uncertainty setting. Values are expressed as percentage differences relative to the unshifted plan. CTV: clinical target volume; CW: chest wall; PTV: planning target volume

The top-ranked VB plan demonstrated the flattest slopes in both D_98%_ and D_2%_, indicating preserved dose stability over a wider range of shifts (Figure 4). SO plans exhibited the steepest deterioration, whereas RO plans demonstrated intermediate robustness. To further quantify motion tolerance, we estimated the isocenter shift required to produce a 3% dose change. The tolerable shift in the VB method varied depending on VB thickness and density, even with the same PTV margin. Several configurations tolerated shifts well beyond the PTV margin, including PTV5mm/VB13mm at 0.4 g/cm³ (9.5 mm), PTV5mm/VB8mm at 0.7 g/cm³ (10.3 mm), and PTV5mm/VB8mm at 1.0 g/cm³ (9.2 mm). Minor ranking reversals occurred between the top two VB configurations; however, the overall trend and the top‐ranked VB configurations remained consistent with the primary robustness ranking. For comparison, SO and RO (5 mm uncertainty) tolerated shifts of 2.4 mm and 5.1 mm, respectively.

Notably, configurations with the same effective VB thickness but different allocations between PTV margin and VB margin demonstrated markedly different tolerable shifts. For example, with a 1.0 g/cm³ density, PTV3mm/VB11mm and PTV8mm/VB11mm both exhibited a total thickness of 11 mm, and the 3% deviation thresholds were 4.5 mm and 11.1 mm, respectively.

## Discussion

This study investigated the robustness of three VMAT planning strategies for PMRT. Among these approaches, the VB method demonstrated the highest level of dosimetric robustness of target coverage, particularly when the VB thickness was set to PTV margin plus 8 mm at 0.4 g/cm³. Under this condition, both D_98%_ and D_2%_ remained within 0.8% for a 5-mm isocenter shift, whereas RO demonstrated moderate robustness and SO exhibited the greatest dose deterioration. These results indicate that appropriate selection of VB parameters can substantially improve target dose robustness compared with conventional approaches under the tested conditions.

These results are consistent with the mechanisms of each approach. VB explicitly promotes fluence beyond the surface, which effectively creates an additional dosimetric margin that maintains target coverage under motion. In contrast, RO primarily preserves dose conformity within predefined uncertainty scenarios and does not inherently promote surface flash unless additional surrogate objectives are introduced, which may explain its intermediate robustness in this setting. SO, which lacks any flash mechanism, is predictably most susceptible to surface motion.

A key finding of this study is that robustness depends on the combined influence of VB thickness and density. Both a thick, low-density VB and a thin, higher-density VB produced favorable performance, indicating differences in flash region size and fluence intensity. These observations indicate that VB-based flash creation cannot be fully characterized by the PTV margin alone. Conversely, certain combinations, including thin VB paired with low density, led to pronounced dose degradation and should be avoided in clinical practice.

Importantly, the most robust VB configuration demonstrated consistent performance across all assessed PTV margins, indicating that its superiority was not dependent on target size. This robustness was observed in both D_98%_ and D_2%_, indicating that the improvement was not limited to preventing underdosage or hotspots but reflected overall target dose stability. Notably, the allocation between the PTV margin and VB margin influenced robustness even when the total VB thickness was identical. This finding indicates that the simple concept of “effective thickness,” defined as the sum of PTV margin and VB margin, may be insufficient, and that robustness is influenced by the combined contribution of PTV margin, VB thickness, and VB density.

These findings refine existing evidence on VB optimization. Previous Eclipse-based studies investigated these parameters separately, with Lizondo et al. focusing on density [3] and Rossi et al. on thickness [13]. Their recommended settings ranked fourth and third, respectively, in our comprehensive analysis. By systematically assessing 27 VB configurations in RayStation, we identified parameter combinations associated with greater robustness of target dose coverage, including PTV margin plus 8 mm at 0.4 g/cm³. Absolute optimal values may differ slightly across treatment planning systems (TPSs); however, the consistency between our results and prior Eclipse findings indicates the broad application of the thickness-density relationship.

RO plans provided better coverage and stability than SO plans, with robustness maintained up to the uncertainty value prescribed during optimization. Specifically, 3% dose deviations occurred at approximately 3.6, 5.1, and 6.9 mm for uncertainty settings of 3, 5, and 8 mm, respectively. These results validate previous observations by Miyasaka et al. and Luo et al. [19,24] and indicate that RO offers a predictable level of target dose robustness. Under the evaluated conditions, the robustness achieved with RO was generally lower than that observed for the top-ranked VB configurations.

Regarding OARs, changes in heart D_mean_ and lung V_20Gy_ under isocenter shifts were small and comparable among SO, VB, and RO for the representative configurations evaluated. Within the evaluated conditions, improved target robustness with VB was not associated with a systematic increase in these OAR dose metrics. Nevertheless, OAR preservation remains an essential consideration in clinical practice, and VB parameter selection should be performed in conjunction with verification of institutional dose constraints.

Direct skin dose metrics were not explicitly evaluated in this study. However, the D_2%_ of CTV_CW, which extends to the near-surface region, was used as a surrogate indicator of potential superficial dose escalation. In the top-ranked VB configurations, CTV_CW D_2%_ remained stable under clinically relevant motion, suggesting that improved target robustness was not accompanied by excessive near-surface hotspots within the evaluated framework. While thin and low-density VB configurations were associated with pronounced D_2%_ escalation and were identified as suboptimal, appropriately configured VB settings did not demonstrate disproportionate increases in near-surface dose. These observations indicate that fluence extension beyond the surface does not inherently translate into excessive superficial dose, although dedicated skin dose evaluation would be required for definitive assessment.

This study has several limitations. First, this is a single-center study using a single TPS, which limits external generalizability. Therefore, the findings require external validation using different TPS platforms to ensure broader applicability. Second, respiratory-induced motion was modeled using purely translational isocenter shifts. Such simplifications do not fully represent the complexity of real breast motion, which predominantly comprises periodic anterior-posterior displacement with amplitudes of typically below 5 mm, as reported in previous studies [4,9]. Rotational components and anatomical deformation were not considered. However, systematic translational perturbations provide a widely interpretable and reproducible framework for robustness assessments across institutions. Third, although non-parametric statistical methods were appropriate for the sample size and study design, more advanced multivariate or mixed-effects modeling may further elucidate potential interactions among planning parameters. Future research is recommended to incorporate 4DCT-based motion modeling, deformable registration techniques, multivariate statistical modeling, expanded OAR and skin dose evaluation, and multi-institutional datasets to further validate and generalize the robustness of VB and RO planning under clinically realistic conditions.

## Conclusions

By systematically evaluating 27 VB and 3 RO configurations, this study addressed the lack of quantitative comparative data on VB and RO in PMRT. We demonstrated that the combined influence of PTV margin, VB thickness, and density is associated with differences in target dose robustness, highlighting parameter dependencies not fully characterized in previous studies. A practical VB configuration with a thickness equal to the PTV margin plus 8 mm at 0.4 g/cm³ achieved the smallest dose changes (<0.8% for a 5-mm shift) and showed greater dosimetric robustness of target coverage than RO under the tested conditions. In contrast, thin, low-density VB settings were associated with pronounced dose degradation and may be suboptimal in clinical practice. Overall, these findings provide a dosimetric reference framework for selecting skin-flash strategies in PMRT, while emphasizing that validation of OAR sparing and clinical outcomes remains necessary before widespread clinical implementation.

## References

[1] Boman E, Rossi M, Haltamo M, Skyttä T, Kapanen M (2016). A new split arc VMAT technique for lymph node positive breast cancer. Phys Med.

[2] Giorgia N, Antonella F, Alessandro C, Eugenio V, Luca C (2011). Planning strategies in volumetric modulated are therapy for breast. Med Phys.

[3] Lizondo M, Latorre-Musoll A, Ribas M, Carrasco P, Espinosa N, Coral A, Jornet N (2019). Pseudo skin flash on VMAT in breast radiotherapy: optimization of virtual bolus thickness and HU values. Phys Med.

[4] Yamauchi R, Mizuno N, Itazawa T, Kawamori J (2020). The influence of respiratory motion on dose distribution in accelerated partial breast irradiation using volumetric modulated arc therapy. Phys Med.

[5] Livi L, Meattini I, Marrazzo L (2015). Accelerated partial breast irradiation using intensity-modulated radiotherapy versus whole breast irradiation: 5-year survival analysis of a phase 3 randomised controlled trial. Eur J Cancer.

[6] Hörner-Rieber J, Forster T, Hommertgen A (2021). Intensity modulated radiation therapy (IMRT) with simultaneously integrated boost shortens treatment time and is noninferior to conventional radiation therapy followed by sequential boost in adjuvant breast cancer treatment: results of a large randomized phase III trial (IMRT-MC2 Trial). Int J Radiat Oncol Biol Phys.

[7] Koivumäki T, Clivio A, Doolan P (2025). The current practice of volumetric modulated arc therapy for breast cancer in Europe - a survey by the EFOMP VMAT breast working group. Phys Med.

[8] Popescu CC, Olivotto IA, Beckham WA (2010). Volumetric modulated arc therapy improves dosimetry and reduces treatment time compared to conventional intensity-modulated radiotherapy for locoregional radiotherapy of left-sided breast cancer and internal mammary nodes. Int J Radiat Oncol Biol Phys.

[9] Kinoshita R, Shimizu S, Taguchi H (2008). Three-dimensional intrafractional motion of breast during tangential breast irradiation monitored with high-sampling frequency using a real-time tumor-tracking radiotherapy system. Int J Radiat Oncol Biol Phys.

[10] Back M, Guerrieri M, Wratten C, Steigler A (2004). Impact of radiation therapy on acute toxicity in breast conservation therapy for early breast cancer. Clin Oncol (R Coll Radiol).

[11] Seppälä J, Vuolukka K, Virén T (2019). Breast deformation during the course of radiotherapy: the need for an additional outer margin. Phys Med.

[12] Rossi M, Boman E, Skyttä T, Haltamo M, Laaksomaa M, Kapanen M (2018). Dosimetric effects of anatomical deformations and positioning errors in VMAT breast radiotherapy. J Appl Clin Med Phys.

[13] Rossi M, Boman E, Kapanen M (2019). Optimal selection of optimization bolus thickness in planning of VMAT breast radiotherapy treatments. Med Dosim.

[14] ICRU Report 83 (2024). ICRU Report 83, prescribing, recording, and reporting intensity-modulated photon-beam therapy (IMRT). Journal of the ICRU.

[15] Fredriksson A, Forsgren A, Hårdemark B (2011). Minimax optimization for handling range and setup uncertainties in proton therapy. Med Phys.

[16] Tomita F, Yamauchi R, Akiyama S, Masuda T, Uchida N, Ishikura S (2025). Evaluating robustness for respiratory motion in accelerated partial breast irradiation using virtual bolus and robust optimization methods. Phys Med.

[17] Fogliata A, Burger H, Groenewald A, Punt L, Parkes J, Cozzi L (2024). Intensity modulated therapy for patients with breast cancer. Practical guidelines and tips for an effective treatment planning strategy. Adv Radiat Oncol.

[18] Yamauchi R, Tomita F, Ishikura S (2025). A robust planning approach for respiratory motion in accelerated partial breast irradiation using volumetric modulated arc therapy. J Radiat Res.

[19] Miyasaka Y, Ono T, Chai H (2024). A robust treatment planning approach for chest motion in postmastectomy chest wall intensity modulated radiation therapy. J Appl Clin Med Phys.

[20] Rossi M, Virén T, Heikkilä J, Seppälä J, Boman E (2021). The robustness of VMAT radiotherapy for breast cancer with tissue deformations. Med Dosim.

[21] He Y, Chen S, Gao X (2023). Robustness of VMAT to setup errors in postmastectomy radiotherapy of left-sided breast cancer: impact of bolus thickness. PLoS One.

[22] Offersen BV, Boersma LJ, Kirkove C (2015). ESTRO consensus guideline on target volume delineation for elective radiation therapy of early stage breast cancer. Radiother Oncol.

[23] (2024). Breast cancer atlas for radiation therapy planning: consensus definitions. http://www.rtog.org/.

[24] Luo H, Lou J, Feng W (2022). Dosimetric advantages of robust optimization combined with flattening filter free in treating cancer of the left breast. Am J Transl Res.

